# Epigenetic crosstalk in chronic infection with HIV-1

**DOI:** 10.1007/s00281-020-00783-3

**Published:** 2020-02-11

**Authors:** Ulrike C Lange, Roxane Verdikt, Amina Ait-Ammar, Carine Van Lint

**Affiliations:** 1 Department of Anesthesiology, University Medical Center Hamburg-Eppendorf, 20246 Hamburg, Germany; 2 Heinrich Pette Institute, Leibniz Institute for Experimental Virology, 20251 Hamburg, Germany; 3 Service of Molecular Virology, Department of Molecular Biology (DBM), Université Libre de Bruxelles (ULB), 6041 Gosselies, Belgium; 4 Institute for Society and Genetics, University of California, Los Angeles, Los Angeles, CA 90095 USA

**Keywords:** HIV-1, Epigenetics, Latency, Latency reversing agents

## Abstract

Human immunodeficiency virus 1 (HIV-1) replicates through the integration of its viral DNA into the genome of human immune target cells. Chronically infected individuals thus carry a genomic burden of virus-derived sequences that persists through antiretroviral therapy. This burden consists of a small fraction of intact, but transcriptionally silenced, i.e. latent, viral genomes and a dominant fraction of defective sequences. Remarkably, all viral-derived sequences are subject to interaction with host cellular physiology at various levels. In this review, we focus on epigenetic aspects of this interaction. We provide a comprehensive overview of how epigenetic mechanisms contribute to establishment and maintenance of HIV-1 gene repression during latency. We furthermore summarize findings indicating that HIV-1 infection leads to changes in the epigenome of target and bystander immune cells. Finally, we discuss how an improved understanding of epigenetic features and mechanisms involved in HIV-1 infection could be exploited for clinical use.

## Introduction

Human immunodeficiency virus type 1 (HIV-1) is a single-stranded positive-sense RNA virus that replicates in CD4^+^ human immune cells. Prerequisite for productive replication is the integration of the reverse transcribed viral DNA into the genome of infected target cells. Integrated HIV-1-derived double-stranded DNA is termed provirus and serves as a template for all viral-derived replication components [1]. In the vast majority of cases, acute infection induces replication-associated cytopathic effects that lead to destruction of the target cells. A small percentage of infected cells however enter a state of latent infection. Such latent HIV-1 infection is characterized by a largely transcriptionally silenced provirus and by the absence of detectable mature viral gene products [2].

Combination antiretroviral therapy (cART) has been developed as a highly efficient treatment to suppress HIV-1 replication at various stages of the viral replication cycle. However, because latency is a non-productive state of infection, latently infected cells escape targeting by cART. They thus contribute to the so-called HIV-1 reservoir, which is responsible for the observed viral rebound in patients upon interruption of antiretroviral treatment. Once infected, individuals can hence never fully clear the virus [3]. This phenomenon has been termed HIV persistence and leads to a state of chronic HIV-1 infection. Around 20 million people worldwide are currently considered chronically HIV-1 infected [4].

HIV-1 proviral DNA in chronic infection shows a number of characteristics. First, an estimated fraction of only around 3% of proviral sequences found in infected individuals on cART is intact and replication competent, as assayed by quantitative viral outgrowth assays [5, 6]. Thus, the vast majority of proviral DNA in chronic infection consists of defective sequences [5, 7–9]. These are thought to arise through the activity of host restriction factors, errors in reverse transcription or RNA splicing and recombination events post integration. While defective proviral sequences do not contribute to the reservoir hurdling HIV-1 cure, they could play a role in pathogenesis of chronic infection. Aberrant transcripts that often harbour translational competence have been reported from proviruses with various defects [7, 9, 10]. These transcripts represent abnormal HIV-RNA species detectable in infected individuals on cART that result from transcription and alternative splicing of integrated proviral DNA with defects such as hypermutations, small internal deletions or mutated major splice donor sites. Although these aberrant transcripts do not support a replicative viral life cycle, they can be translated to viral proteins such as gag or give rise to novel chimeric HIV-protein species. These proteins have been shown to be capable of eliciting cytotoxic T-lymphocytic (CTL) responses and thus postulated to induce immune activation [9, 10]. Chronic HIV-1 infection is therefore characterized by presence of a proviral-derived DNA burden integrated within the genomes of infected target cells. This burden is made up of a percentage of replication-competent intact proviruses as well as a large fraction of defective HIV-1-derived sequences.

The pathogenic impact of HIV-1 proviral-derived DNA burden is likely dependent on the regulation of the viral sequences. Thus, we need to gain a comprehensive understanding of (i) molecular pathways that regulate HIV-1-derived DNA and (ii) the mechanisms that influence genomic crosstalk between proviral DNA and the human target genome. This review will focus on the epigenetic aspects involved in these processes.

The term epigenetics has originally been introduced to describe heritable features in cell identity and physiology that are not linked to alteration in genetic sequence composition [11]. Today, the field of epigenetics investigates a broad variety of mechanisms that collectively affect DNA-dependent activities [12]. Of predominant interest have been epigenetic mechanisms that determine transcriptional state and activities of gene expression. Chromatin, i.e. the totality of DNA-associated proteins and RNAs, is considered the platform through which epigenetic regulation is exerted [13, 14]. This regulation is driven through chemical modifications of DNA or chromatin components or alterations in chromatin composition, which lead to structural changes that influence accessibility of regulatory DNA regions to for example transcriptional regulators or effector proteins. Furthermore, these modifications often serve as recruitment platforms for binding of specific downstream effectors. The most studied and first described epigenetic chromatin features are covalent modifications of DNA bases and histone proteins. These include cytosine methylation of CpG dinucleotides (5mCpG) in DNA and post-translational modifications (PTMs) of mainly N-terminal histone residues, such as methylation (me), acetylation (ac) or phosphorylation (phospho) [15, 16]. It has become evident that certain epigenetic features are usually associated with either a transcriptionally active and open or transcriptionally silent and condensed chromatin state, termed euchromatin or heterochromatin, respectively [13, 14]. Unmethylated DNA, hyper-acetylated and hypo-methylated histones located on promoter *cis*-regulatory elements are generally markers for euchromatin, whereas methylated DNA, hypo-acetylated and hyper-methylated histones are considered heterochromatin features. This is however a very simplistic representation, since the position of one and the same PTM within a histone can be associated with opposing activity states. For example, trimethylation of lysine 4 in histone 3 (H3K4me3) generally marks active promoter regions, while trimethylation of lysine 9 or trimethylation of lysine 27 in histone 3 (H3K9me3, H3K27me3) demarcates a transcriptionally repressed promoter. A large pool of epigenetic effector proteins has been described, which are responsible for catalysing or indeed removing specific modifications, such as DNA methyltransferases (DNMTs), histone methyltransferases (HMTs) or histone acetyltransferases (HATs) for example. Overall, it is today evident that epigenetic regulation is mediated by a dynamic and highly complex interplay of different epigenetic marks and pathways that involve DNA, chromatin components and in addition also higher-level features, such as for example 3D nuclear organization [13, 14, 17, 18].

A multitude of studies focusing on epigenetic mechanisms in HIV-1 biology and in particular HIV-1 persistence was initiated following the observation that HIV-1 latency contributes to the establishment of a reservoir that prohibits viral elimination and thus a cure for HIV-1 infection. When reviewing findings from these and ongoing studies, two points should be noted. First, our current understanding of how proviral activity is epigenetically regulated is largely based on analysis of cellular HIV-1 infection models. This is due to the relative paucity, lack of molecular markers and heterogeneous identity of latently HIV-1 infected cells in individuals on cART, which largely prevents analysis of regulatory mechanisms in vivo or on patient-derived materials. Secondly, also as a result of this technical hurdle, work was so far mainly focused on understanding how epigenetic mechanisms influence integrated HIV-1 proviral DNA sequences. There are few studies to date that have investigated epigenetic effects of acute or latent HIV-1 infection on the host genome as a result of proviral/human DNA crosstalk.

## Chromatin landscape of the HIV-1 provirus and its role in the control of viral gene expression

Proviral HIV-1 double-stranded DNA, once integrated into the host genome, is indissociable from cellular genes. As such, it adopts a chromatin architecture. Since HIV-1 exploits the cellular machinery for transcription of its own genes, the dynamic changes in proviral chromatin architecture, especially at the 5′ LTR that contains the viral promoter, are crucial for HIV-1 gene regulation.

### Proviral chromatin architecture and the viral protein Tat as a key modulator for HIV-1 transcription

**Fig. 1:**
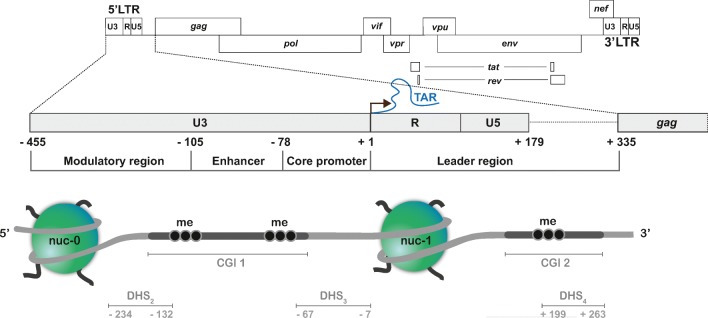
Organization of the HIV-1 5′ long terminal repeat (LTR). Schematic representation of HIV-1 genome structure (above) and organization and nucleosome positioning at the 5′ LTR (below). The 5′ LTR is divided in four functional domains: modulatory region, enhancer, core promoter and leader region. Transcriptional start site (TSS; black arrow) and TAR element are marked. The LTR is embedded into two nucleosomes nuc-0 and nuc-1. DNase I hypersensitive sites are demarcated (DHS_2_, DHS_3_ and DHS_4_). Two CpG islands (CGIs) surround the HIV-1 TSS. Numbers indicate base pair positions relative to the TSS (position + 1)

To decipher the chromatin architecture of the HIV-1 provirus, early experiments focused on mapping nucleosome deposition by measuring sensitivity to Dnase I and to micrococcal nuclease (MNase) digestion. These experiments have shown that, regardless of the integration site in the host genome, nucleosomes are strictly deposited at specific positions in the HIV-1 provirus (Fig. 1) [19–21]. In particular, the 5′ LTR is embedded into two distinct nucleosomes (called nuc-0 and nuc-1), separated by the two DNase I hypersensitive sites DHS_2_ and DHS_3_ (Fig. 1) [20]. Mechanistically, the 5′ LTR nuc-1 is actively positioned in a refractory sequence immediately downstream of the HIV-1 transcription start site (TSS) by the cellular ATP-dependent chromatin remodelling BAF complex [20, 21]. In this position, nuc-1 causes pausing of cellular RNA polymerase II (RNAPII). It thus constitutes a repressive barrier to the progression of the cellular transcription machinery [20, 21]. As a consequence, while recruitment of cellular transcription factors (TFs) and their associated coactivators to the HIV-1 5′ LTR is sufficient to trigger the initiation of viral transcription, the key rate-limiting step in HIV-1 gene expression is transcriptional elongation [22, 23].

**Fig. 2:**
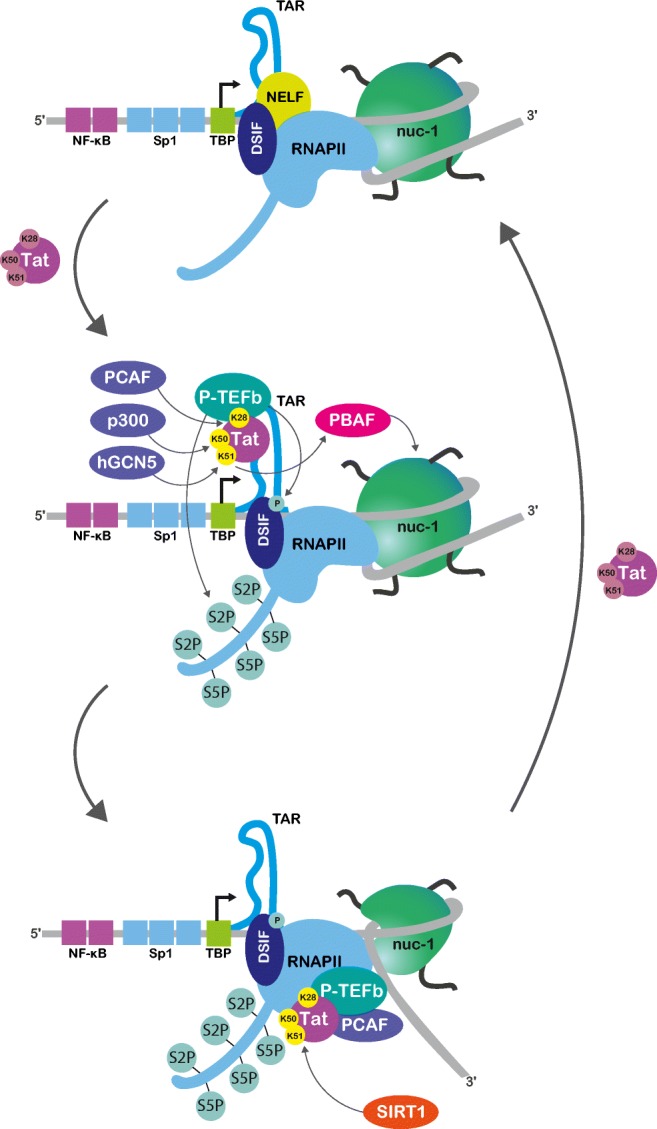
Tat-mediated transactivation of HIV-1 transcription. In the absence of Tat, binding of cellular TFs to the 5′ LTR is sufficient to initiate transcription. However, RNAPII pauses after the synthesis of few abortive transcripts due to the recruitment of NELF and DSIF. As soon as Tat accumulates in the nucleus, it binds the TAR element and promotes RNAPII transition to processive elongation. Tat acetylation by PCAF on K28 allows P-TEFb recruitment and Cdk9-induced phosphorylation of RNAPII CTD tail. Cdk9 also phosphorylates DSIF and NELF, transforming the first in a positive elongation factor and releasing the second. Acetylation of Tat on K50 by p300 facilitates P-TEFb release and recruits PBAF that remodels the repressive nuc-1. Acetylation of Tat on K51 by hGCN5 favours Tat transfer to the elongating RNAPII. At the end of transcription, Tat is deacetylated by SIRT1, allowing its recycling in a new feedback loop. Adapted from [27]

To overcome this elongation block, HIV-1 employs the virally encoded Tat transactivator (Fig. 2). Tat initiates a cascade of events that ends in disruption of nuc-1 and remodelling of the chromatin structure of the 5′ LTR [22]. Briefly, the Tat transactivator binds the TAR element, a 60-nt stem-loop structure located at the 5′ end of all nascent HIV-1 transcripts. This provokes the recruitment of the cellular positive transcription elongation factor B (P-TEFb), composed of the cyclin-dependent kinase Cdk9 and cyclins T [24]. Together with other factors of a super elongation complex (SEC) [25], the kinase component of P-TEFb directly targets RNAPII. RNAPII pausing is therefore overcome and a productive form of HIV-1 transcription is resumed. Importantly, this positive regulatory circuit centered around Tat transactivation is strongly regulated by Tat post-translational modifications [22]. Tat acetylation on its lysine 28 (K28) residue by PCAF (p300/CBP associated factor) promotes P-TEFb recruitment [26], whereas acetylation on the lysine 50 residue (K50) by p300/CBP and the lysine 51 residue (K51) by hGCN5 facilitates release of P-TEFb and transfer of Tat from the TAR element to the elongation RNAPII complex, respectively [27]. At the end of the elongation process, Tat deacetylation by the histone deacetylase (HDAC) sirtuin 1 (SIRT1) allows its dissociation from RNAPII and PCAF and its recycling to initiate a new transactivation feedback loop (Fig. 2) [27].

Tat post-translational modifications thus determine its different roles in transactivation. Importantly they furthermore also serve for the recruitment of cellular epigenetic modifiers [27, 28]. Indeed, PCAF, p300/CBP and hGCN5 are well-characterized HATs that not only acetylate Tat but are also associated with extensive acetylation of histones H3 and H4. This acetylation contributes to the establishment of a more accessible chromatin environment in the vicinity of the 5′ LTR and thus favours transcription of HIV-1 genes [22, 28]. Furthermore, acetylation on Tat K50 causes recruitment of PBAF that actively remodels nuc-1 and thereby allows processive elongation [21].

### Epigenetic control of HIV-1 latency

The transcriptional competence of the HIV-1 provirus is heavily controlled at the epigenetic level through exploitation of cellular enzymes. During HIV-1 latency, transcriptional silencing of viral gene expression is maintained by numerous interrelated epigenetic mechanisms. These include histone PTMs, modification of the DNA itself, as well as non-coding RNA (ncRNA)-mediated epigenetic mechanisms. Recently, considerable attention has also been paid to the role of the chromatin state at the site of proviral integration as means to control HIV-1 expression. Of note, while we will here focus on the epigenetic control of HIV-1 latency, proviral silencing is maintained along the entire spectrum of the gene expression pathway, including at post-transcriptional and translational levels [29].

#### Histone PTMs in the control of HIV-1 latency

**Fig. 3:**
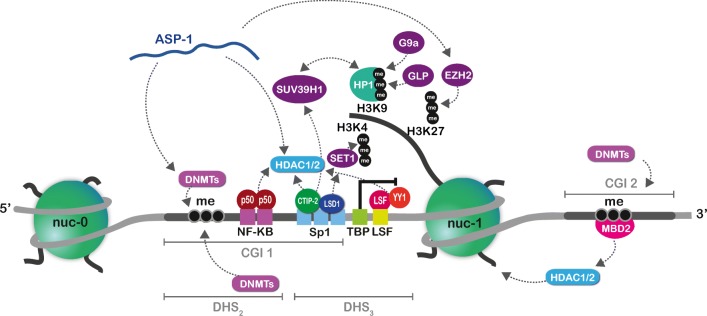
Epigenetic control of HIV-1 silencing during latency. HIV-1 silencing during latency is regulated through epigenetic mechanisms. During latency, transcription factors redundantly recruit histone modifiers. For instance, the negatively acting NF-κB homodimer p50-p50 occupies cognate binding sites in the 5′ LTR and recruits HDAC1 and HDAC2. In microglial cells, the cellular factor CTIP2 represses HIV-1 gene expression at least through three distinct modes. One of them is depicted in the figure. CTIP2 and LSD1 bind the Sp1 sites in the 5′ LTR. CTIP2 sequentially recruits HDACs and the HMT SUV39H1 that catalyses H3K9me3. This mark is recognized by HP1 that recruits further SUV39H1 units spreading the heterochromatic H3K9me3 mark. In parallel, LSD1 recruits the hCOMPASS complex, notably containing the histone methyltransferase (HMT) SET1 that stimulates H3K4me3. Furthermore, several HMTs (including G9a, GLP and EZH2) are responsible for depositing H3K9me2/3 and H3K27me3, respectively; their mode of recruitment to the HIV-1 promoter remains unclear. Two CpG islands (CGIs) surround the HIV-1 TSS and are heavily methylated by DNA methyltransferases (DNMTs), allowing the recruitment of MBD2 and the associated NuRD complex (containing HDAC2) to the second CGI. Recent reports show that the HIV-1-encoded antisense transcript ASP-1 also contributes to epigenetic silencing through promoting recruitment of Dnmt3, HDAC1 and EZH2 to the 5′ LTR

In addition to nucleosome positioning discussed above, the involvement of histone tail PTMs in HIV-1 latency has been extensively studied [27, 30]. A wide variety of LTR-binding cellular TFs act as transcriptional repressors through redundant recruitment of cofactors and induction of epigenetic silencing [27, 31]. For instance, during latency, NF-κB binding sites in the 5′ LTR are occupied by the negatively acting NF-κB homodimer p50-p50 that recruits class-I histone deacetylase 1 (HDAC1) and establishes a heterochromatin environment at the viral promoter [32]. Similarly, HDAC1 is also recruited cooperatively by the host factors YY1 and LSF (Fig. 3) [33, 34]. This redundant recruitment of one and the same HDAC, as well as the recruitment of different classes of HDACs by several independent mechanisms to the 5′ LTR during latency, explains the success of using pan-HDAC inhibitors (HDACi) in HIV-1 latency reversing strategies [30, 35].

In addition to cumulative recruitment of one type of histone modifier, some repressive cellular TFs also bridge different histone modifications. This is the case for CTIP2 (COUP—TF interacting protein 2)/BCL11B. In microglial cells, CTIP2 mediates at least one mechanism of HIV-1 gene repression through recruitment of a multi-enzymatic chromatin-modifying complex that establishes a heterochromatic environment at the 5′ LTR, in a Tat-independent manner (Fig. 3) [36, 37]. Indeed, in these cells, CTIP2 is physically recruited to the HIV-1 promoter by its interaction with Sp1 bound to the GC-boxes of the 5′ LTR [38]. This recruitment is made possible by histone lysine-specific demethylase 1 (LSD1) also bound to Sp1 sites [39]. LSD1 recruits the multicomponent COMPASS complex that ultimately leads to the accumulation of H3K4me3. Exceptionally, in the context of the HIV-1 promoter, this accumulation is associated with transcriptional repression [39]. In parallel, CTIP2 sequentially recruits HDAC1/2 to deacetylate H3 and the histone methyltransferase (HMT) SUV39H1 (suppressor of variegation 3-9 homolog 1) that promotes H3K9me3 [36]. Through its chromodomain, heterochromatin protein 1 (HP1) recognizes H3K9me3, which leads to further recruitment of SUV39H1. In this way, heterochromatin spreads along the HIV-1 promoter further stabilizing a state of latency [36].

Finally, interdependent recruitment of histone modifiers is also postulated to occur during HIV-1 latency. For instance, HMT EZH2, together with the EZH2-mediated H3K27me3 mark, have been reported at the viral promoter in both cell lines and primary cell models for HIV-1 latency (Fig. 3) [40]. While the mode of EZH2 recruitment to the latent 5′ LTR is still unclear, it could be functionally linked to other epigenetic marks, especially to H3K9me2/3, as reported in embryonic stem cells [41]. Accordingly, both EHMT1 (euchromatin histone methyltransferase)/GLP and EHMT2/G9a, which participate in HIV-1 latency by depositing H3K9me2 on the HIV-1 promoter in latently infected T cell lines [42–44], could be cooperatively recruiting EZH2.

#### DNA methylation in the control of HIV-1 latency

DNA methylation is a post-synthetic, reversible and hereditary epigenetic mark that in mammals occurs predominantly on cytosine residues in the context of CpG dinucleotides [45, 46]. Mechanistically, DNA methylation of CpG dense regions at gene promoters, termed CpG islands (CGIs), is in general associated with repression of transcription, either directly or indirectly [47]. DNA methylation-mediated repression of viral promoters has been involved in latency of several viruses [48], including the bovine leukaemia virus (BLV) and the human T-lymphotropic virus type 1 (HTLV-1) retroviruses [49, 50]. Early reports already established that DNA methylation modulates HIV-1 gene expression [51, 52]. Two CGIs surround the HIV-1 TSS (Fig. 1) and compelling evidence shows that these two CGIs are hypermethylated in model T cell lines and in primary cell models for HIV-1 latency [53–55]. Furthermore, in latently infected cell line models, hypermethylation of the second HIV-1 promoter CGI provokes recruitment of the Methyl-CpG Binding Domain Protein 2 (MBD2). MBD2 is associated with the chromatin-modifying complex NuRD that also contains HDAC2 (Fig. 3) [53].

Thus, DNA methylation is intimately linked to other epigenetic modifications and participates in the heterochromatic silencing of the 5′ LTR in cellular models of latent infection. However, primary cells from HIV^+^ individuals have been found to be more heterogeneous concerning the DNA methylation status of the viral promoter. Some studies showed an accumulation of HIV-1 promoter methylation [53, 54, 56], whereas others disputed the existence or the relative contribution of HIV-1 promoter methylation to viral persistence [6, 57, 58]. The heterogeneous profiles reported by these studies are likely due to several reasons. These include technical variations in sample analysis, variations in the nature of the provirus and variations in the clinical characteristics of the patients. For instance, one study showed that accumulation of methylation was especially low at the promoter of replication-defective proviruses [6]. This might suggest differential epigenetic mechanisms of HIV-1 repression in replication-competent and replication-defective reservoirs of HIV-1. Furthermore, the use of PCR-based methods to analyse methylation profiles, using bisulfite conversion coupled with sequencing, is inherently more difficult in clinical samples [59, 60]. Finally, clinical characteristics such as duration of infection [56] and duration of antiretroviral therapy treatment [61, 62] were both shown to positively associate with the accumulation of HIV-1 promoter methylation. Further understanding of the recruitment modes and modalities of DNMTs to the HIV-1 promoter will likely provide more insights into the current discrepant reports.

#### The contribution of ncRNAs to HIV-1 latency

Different classes of ncRNAs have been involved in epigenetic control of gene expression through the recurrent formation of RNA scaffolds that serve to recruit chromatin-modifying complexes [63]. Regulatory ncRNAs can be classically classified according to their size, with transcripts longer than 200 nt termed long ncRNAs (lncRNAs). Smaller transcripts include microRNAs (miRNAs) or piwi-interacting RNAs (piRNAs) [64]. In mammals, gene expression by small ncRNAs involves little chromatin structure regulation, with the exception of certain piRNAs that promote methylation of transposons and thereby suppress their activity during spermatogenesis [63, 64]. On the contrary, multiple studies have demonstrated a role for specific lncRNAs in controlling gene expression through modelling of chromatin structure in homeostatic biological processes as well as diseases [65]. As for HIV-1 latency, several cellular lncRNAs have been shown to promote latency, directly or indirectly. One example is the lncRNA NRON that indirectly restricts HIV-1 gene expression by inducing Tat proteasomal degradation [66].

It has been debated whether HIV-1 produces ncRNAs. Some reports suggest that miRNAs can be derived from certain secondary RNA structures of the HIV-1 genome [67]. Furthermore, the ability of HIV-1 to transcribe its genome in antisense from the 3′ LTR has long been suspected [68]. Indeed, both LTRs can function as transcriptional promoters, even though the 5′ LTR initiates transcription more often, which is believed to occlude transcription from the 3′ LTR [69, 70]. Many studies have since confirmed the expression of an antisense transcript that encodes the HIV-1 antisense protein (ASP-1) from a reading frame located on the negative strand in the *env* gene [70–72]. While the function of the ASP-1 protein remains unclear, two reports have recently highlighted that the *ASP-1* RNA is associated with epigenetic regulation of the 5′ LTR in a similar manner to that of cellular lncRNAs [73, 74]. It was shown that downregulation of the *ASP-1* transcript was associated with decreased recruitment of DNMT3a, HDAC1 and EZH2 to the 5′ LTR, implicating *ASP-1* in HIV-1 epigenetic silencing [73]. In accordance, it was recently found that *ASP-1* RNA recruits EZH2 to the 5′ LTR, which provokes placement of the repressive H3K27me3 mark, nuc-1 assembly and transcriptional silencing (Fig. 3). *ASP-1* thus promotes viral latency [74]. Altogether, the HIV-1-encoded antisense transcript appears to branch several epigenetic processes in maintaining a heterochromatic environment at the HIV-1 5′ LTR during latency.

#### Integration site–dependent regulation of proviral HIV-1 DNA

HIV-1 preferentially integrates within transcriptionally active genes and within regions bearing enhancer marks [75–77]. Indeed, HIV-1 integration is controlled by cooperating viral and cellular determinants, such as the cellular cofactor LEDGF/p75 that recognizes H3K36me3 marks for targeted HIV-1 integration [78, 79]. In this euchromatin context, HIV-1 silencing may seem counter-intuitive and a highly discussed open question is how the chromatin environment at the integration site dictates heterochromatinization of the HIV-1 provirus. Two phenomena that have been observed in HIV-1 infected individuals on cART are remarkable in their suggestion of a functional crosstalk between proviral-derived sequences and the human genome at the site of proviral integration. First, chronically infected individuals present genomic hotspots or recurrent integration genes, where proviral-derived sequences are preferentially found [80–84]. This results from a reshaping of the initial integration site bias in acute HIV-1 infection, which is determined by a number of genetic, epigenetic and mechanistic features [8, 85]. Second, a subset of proviral integration sites observed in chronic HIV-1 infection appears linked to clonal expansion of the targeted cell [81–83, 86–88]. Such clonally expanded cells have been found to carry intact as well as defective proviral sequences and appear to be present in most studied cases of HIV-infected individuals on cART [86, 89–91]. The mechanisms underlying clonal expansion are to date elusive. Expansion mediated by antigen- and cytokine-driven proliferation, a well-known phenomenon in T cell biology, has been discussed [8, 87, 92]. Alternatively, there is increasing evidence that the genomic locus at the proviral integration site and hence a functional proviral/human DNA crosstalk could play a dominant role. Several studies have shown that the genomic context influences HIV-1 proviral expression and inducibility [77, 93–98]. Recurrently found gene loci in chronic infection have been proposed to offer a genetic and epigenetic environment that promotes transcriptionally silent persistence of proviral genomes and therefore maintenance of the reservoir [99]. On the other hand, proviral-derived sequences themselves could alter expression of genes located nearby. Chimeric proviral/human transcripts that arise from exaptation of the HIV-1 LTR promoter region for transcription of human endogenous gene products have indeed be observed [89, 100]. In this way, proviral-derived DNA impacts on the host cell transcriptome and influences host cell physiology and behaviour such as differentiation, proliferation and/or survival and thereby stimulates expansion of the host cell clone [8, 82, 83, 85, 87]. This scenario could also explain observed clonal proliferation of cells with largely defective proviruses and solo-LTRs that are transcription/translation incompetent, cannot elicit immune responses and therefore are unlikely to undergo antigen-driven expansion [6, 81]. In this context, it is remarkable that a number of recurrent integration sites found in chronic infection are in gene loci associated with proliferative control, cell differentiation or oncogenesis [82–84, 89].

Thus, while observations in HIV-1 chronically infected individuals point towards the importance of a functional crosstalk between the HIV-1 provirus and its environment of integration, the contribution of epigenetic mechanisms to this crosstalk (i.e. in terms of hotspots of integration and of clonal expansion) still needs to be further clarified.

### Epigenetic regulation of HIV-1 latency establishment

Fuelled by therapeutic considerations, much attention has been paid on how HIV-1 latency is epigenetically maintained rather than how it is effectively established. Mathematic modelling has emphasized that stochastic fluctuations of Tat levels might act as a molecular switch in driving initial HIV-1 latency [101], independent of the activation state of infected cells [102]. Indeed, the absence of Tat prevents epigenetic remodelling of the 5′ LTR, especially of the repressive nuc-1, and this contributes to maintenance of a heterochromatic structure at the HIV-1 promoter [103]. Still, few studies have addressed the epigenetic mechanisms involved in HIV-1 latency establishment. This is mainly due to technical limitations: the vast majority of current epigenetic profiling techniques interrogates biological processes in a static rather than a dynamic manner [104]. In addition and as mentioned before, most mechanistic studies are performed in artificial models for HIV-1 latency since work on primary patient-derived materials is very cumbersome and, especially in the context of functional assays, this work is often not possible [105].

In a study focused on the kinetics of HIV-1 latency establishment, it has been found that HIV-1 latency is heterogeneously established in cell populations, either by immediate silencing or by continuous decline in HIV-1 gene expression [106]. While H3K9me3 accumulation on the 5′ LTR was not different between the two cellular populations, 5′ LTRs that underwent immediate silencing initially presented higher levels of H3K27me3 [106]. Interestingly, also in the time-dependent silenced population, H3K27me3 accumulated at the LTR region [106]. This finding indicates a crosstalk between the H3K9me3 and H3K27me3 marks that appears important for latency establishment. Nucleosome positioning and histone PTMs are therefore good candidates for early events leading to HIV-1 latency. On the contrary, DNA methylation has been suggested to be a late event. Indeed, observations from various fields of science support the view that DNA methylation locks gene expression long-term rather than initiating heterochromatic silencing [107, 108]. In accordance, reports highlighting the temporal dimension in accumulation of 5mCpG on 5′ LTR sequences in HIV-1-infected patients [56, 61] suggest that this epigenetic mark does likely not contribute to epigenetic establishment of HIV-1 latency. Furthermore, cellular lncRNAs might be an early incentive in HIV-1 silencing. Indeed, it is known that HIV-1 infection is associated with changes in the landscape of ncRNAs in infected cells [109]. These HIV-1-induced variations, as well as natural variations of ncRNAs profiles among individuals [110, 111], could thus constitute stimuli for establishing an HIV-1 latency state. While a functional lncRNome-wide interrogation of HIV-1 gene expression modulation is currently still lacking, future research in this area holds the promise to further our understanding of epigenetic features underlying HIV-1 latency establishment. Finally, it is likely that depending on the integration site, different epigenetic mechanisms of latency establishment might be at play [77, 112].

## Changes of the cellular epigenome as a result of HIV-1 infection

HIV-1 gene expression, as previously described, is actively controlled through usage of the epigenetic machinery of infected host cells. While much data has been gathered during the past decades to decipher details of this interaction, we are currently only beginning to understand converse effects of HIV-1 infection, i.e. how host cell epigenomes and the epigenomes of bystander immune cells are altered in response to HIV-1 infection.

There are a number of possibilities for how such alterations could be mediated, as examples of different viral infections have shown [113]. First, viral proteins might inherently possess enzymatic activities of epigenetic modifiers. This has for example been demonstrated for vSET, a protein of *Paramecium bursaria* chlorella virus (PBCV-1), which codes for a viral SET domain enzyme that catalyses deposition of the repressive H3K27me3 mark on host cell chromatin [114]. Second, viral-encoded proteins could interact with epigenetic players of infected cells and thereby alter their activity. This results in a changed epigenomic profile of infected cells, which could, for example, promote viral replication, a state of viral latency or indeed alter the proliferative behaviour of targeted cells. Examples for such scenario are found during infection with human gamma herpesviruses Kaposi’s sarcoma-associated virus (KSHV) and Epstein-Barr virus (EBV) [113]. The KSHV-encoded latency-associated nuclear antigen (LANA) interacts with SUV39H1 and DNMT3A to induce transcriptional repression of a range of host genes [115, 116]. These changes have been proposed to induce epigenetic reprogramming of infected cells, which can be associated with transition towards a transformed phenotype and explain KSHV-associated tumour development [113]. In a similar manner, various EBV-encoded nuclear antigen (EBNA) proteins have been shown to functionally interact with the cellular polycomb epigenetic repression complex to induce transcriptional downregulation of tumour suppressor genes in infected cells [113, 117]. As a third scenario, cellular epigenetic profiles could be altered as an indirect consequence of viral infection. Innate cellular mechanisms sense viral infection and initiate signalling cascades that eventually result in epigenomic restructuring. In this case, epigenetic changes might not only be seen in infected target cells but bystander cells might equally be affected through infection-induced cytokine signalling and an altered microenvironment. Through this mechanism, chronic inflammation induced by hepatitis viruses HBV and HCV has been proposed to result in aberrant DNA methylation signatures in hepatocytes of infected livers [118, 119].

In the case of HIV-1 infection, few studies have so far focused on epigenetic changes on the host genome and further mechanistic insights are yet to be uncovered. These studies primarily addressed the cellular methylome, i.e. alterations in the extent and pattern of 5mCpG in cellular genes in response to HIV-1 infection. Notably, the analysis was mainly undertaken in CD4^+^ T cells or peripheral blood mononuclear cells (PBMCs) of HIV-1-infected individuals on ART, which included infected, but to a large extent also bystander, non-infected cells. At least in part, observed effects are thus likely indirect effects of HIV-1 infection. A pioneering report two decades ago also showed that HIV-1 infection results in increased DNMT activity and de novo methylation of a single CpG in the gamma interferon (IFN-γ) promoter [120]. This provoked transcriptional downregulation of the cytokine as important player for different immune functions [120]. The same team further showed that HIV-1 infection was associated with hypermethylation and reduced expression of p16^INK4A^, a tumour suppressor gene [121]. These findings brought about the idea that aberrant DNA methylation might be a conserved mechanism of HIV-1 pathogenesis. Indeed, more recently, the use of array-based genome-wide techniques for methylome analysis has revealed that blood cells of HIV-1-infected individuals on ART are epigenetically altered in a characteristic way, linking HIV-1 infection to premature ageing and abnormal immune responses [122–124]. One study compared methylation patterns at over 26,000 genome-wide CpG sites validated as ageing markers and came to the conclusion that HIV-1 infected individuals on cART showed an average epigenetic ageing advancement of 4.9 years compared with healthy controls [124]. The authors furthermore observed global deregulation of the methylome across over 80,000 CpG sites, which in addition to changes reminiscent of advanced age, also showed local abnormalities specific for HIV-1 infection. These include hypomethylation at the human leukocyte antigen (HLA) locus, which indeed could suggest epigenetic regulation in innate immune responses involved in HIV-1 infection control [124]. Interestingly, it appears that antiretroviral therapy can alter observed methylome changes and thus might influence premature ageing as well as onset and progression of comorbidities in HIV-1 infected individuals [123].

Although much remains to be analysed, these studies collectively support the early finding that HIV-1 infection profoundly alters the cellular methylome. Based on our understanding of epigenetic regulation as a complex interplay of different features, it would be astonishing if methylome changes would not be accompanied by genome-wide changes of further epigenetic marks. Indeed, there is preliminary data that HIV-1 infection also alters levels of several histone PTMs [125, 126]. One study showed that HIV-1 infection ex vivo is accompanied by strong fluctuations in histone PTM levels as demonstrated by mass spectrometry and transcriptional profiling of PTM-associated enzymes [126]. In accordance with the findings on increased CpG methylation upon infection, a second report suggested that global repressive histone marks, such as H3K9me3 and H3K27me3 increase [125]. Notably, these studies focused on acute infection ex vivo—data on global histone PTM changes in chronically HIV-infected individuals has to our knowledge not yet been reported. This will likely change in the near future with current advances in sequencing technologies and the fast evolving protocols for genome-wide histone PTM analyses on low cell numbers in primary cells.

In conclusion, current pioneer works indicate that HIV-1 infection leads to long-term epigenetic reprogramming of target and bystander immune cells. While underlying molecular mechanisms remain to be uncovered, this reprogramming could play an important role not only in promoting HIV-1 persistence but also in the development of chronic HIV-1 disease and associated comorbidities.

## Epigenetic targets in clinical approaches to HIV-1 disease

The realization that cART, although efficiently controlling viral replication, could not eliminate HIV-1 from infected individuals has initiated a now decade-long search for clinical strategies to achieve an HIV-1 cure. Efforts have mainly been focused on targeting the latent HIV-1 reservoir responsible for viral persistence. In addition, much work has been done to find approaches to strengthen immunological defences to HIV-1, such as for example through use of anti-HIV-1 broadly neutralizing antibodies (bNAbs) or chimeric antigen receptor (CAR) T cells targeted to HIV-1-infected cells [127]. A second avenue of research has focused on counteracting the state of HIV-1 latency. This ‘shock and kill’ approach has been based on the idea that forced reversal of proviral transcriptional repression (‘shock’) would lead to fast depletion of viral reservoir cells, while ongoing cART would prevent new reservoir seeding and immunological defences would clear reactivated reservoir cells (‘kill’) [1]. Much effort has therefore been spent on finding so-called latency reversing agents (LRAs), i.e. compounds that have the capacity to induce proviral transcription from silenced HIV-1 5′ LTR [128].

The first LRA compounds tested were the powerful immune-activating interleukin-2 (IL-2) [129] and anti-CD3 antibodies [130], based on the observation that engagement of the T cell receptor consistently activated HIV-1 production in latently infected CD4^+^ T cells [35]. However, cART discontinuation after treatment with these antibodies resulted in rapid plasma viral rebound in all patients [131]. Thanks to our improved understanding of molecular mechanisms underlying HIV-1 latency, a variety of different LRA classes has since been developed (reviewed in detail in [128]). Of particular clinical interest are a range of so-called epi-LRAs, i.e. agents that reverse proviral latency through direct interference with epigenetic silencing mechanisms at the 5′ LTR [35]. These include inhibitors of histone deacetylases (HDACi), e.g. Vorinostat and Panobinostat, inhibitors of histone methyltransferases (HMTi), e.g. Chaetocin, and inhibitors of DNA methylation (DNMTi), e.g. 5-aza-2′-deoxycytidine [35]. Since epi-LRAs performed well in activation of latent HIV-1 ex vivo and importantly in a number of cases, these compounds have already been FDA-approved for use in clinical practice in the context of anti-cancer regimens, several trials have been undertaken to investigate their potential in purging the HIV-1 reservoir in chronically infected individuals. However, although transient HIV-1 production has been observed, no trials using individual LRA have so far succeeded in significantly reducing HIV-1 reservoir size [132–134]. One possible reason for these findings is the growing evidence that HIV-reservoirs are of highly heterogeneous nature, not only regarding cellular identities but also concerning cellular activation state and tissue type-dependent microenvironment [135–137]. Therefore, combination of epi-LRAs with LRAs targeting different cellular pathways, such as for example protein kinase C (PKC) agonists or positive elongation factor B (P-TEFb)-releasing agents will likely be necessary for optimal HIV-1 latency reversal in vivo [128]. A possibly even greater challenge in the ‘shock and kill’ approach is the achievement of a sufficient ‘kill’ of targeted reservoir cells. It has become evident that intricate adjuvant immunotherapies will be required to eliminate newly activated cells and prevent proliferation of the reservoir [138, 139]. These hurdles have currently somewhat halted the surge for ‘shock and kill’ in HIV cure research and have allowed for alternative concepts to be brought forward.

One such concept is the ‘block and lock’ strategy—an approach which promotes the idea of disarming HIV-1 reservoir cells through blocking HIV-1 transcriptional activity and locking the proviral promoter in a state of deep latency [140]. This concept not only opposes the strategy of ‘shock and kill’, it furthermore also complies with a growing perception in the field that full elimination of HIV-1 might clinically not be achievable. Instead, therapeutic efforts should support a sustainable remission, i.e. a state in which HIV-1-infected individuals are able to control the viral burden without need for continuous cART. Since at its core, transcriptional activity of the HIV-1 provirus is regulated through epigenetic mechanisms, strategies for deep latency will need to target the proviral epigenetic landscape for long-term, heritable silencing. This has indeed been found for the most promising ‘block and lock’ agent so far reported, the Tat inhibitor didehydro-cortistatin A (dCA) [141–144]. dCA is an analog of the natural steroidal alkaloid cortistatin A, which prevents Tat/TAR interaction and thus Tat-mediated transactivation of HIV-1 promoter through binding the TAR-binding domain of Tat. In cellular HIV-1 latency models, treatment with dCA promotes heterochromatinization of the HIV-1 5′ LTR, with an increase of deacetylated H3 at nuc-1 and the recruitment of repressive chromatin-modifying complexes to the HIV-1 promoter [143]. In CD4^+^, T cells of HIV-1 positive individuals dCA thus appear to induce a state of persistent latency—HIV-1 transcriptional activity is blocked and increasingly becomes refractory to reactivation by LRAs [144]. This finding has been mimicked in a mouse model for HIV latency, where dCA treatment reduced tissue HIV-1 RNA and although viral rebound upon discontinuation of ART was still observed, rebound was delayed and quantitatively reduced [141]. Clinical trials with dCA in humans have so far not been reported and several questions, including potential viral mutation-based drug resistances remain to be addressed [145, 146]. Nevertheless, the findings on dCA show that inhibitors of Tat or indeed agents that mediate heterochromatinization of the proviral LTR might in future be important components of clinical approaches for sustainable remission in HIV-1 disease.

In this context, a second class of compounds, the so-called LEDGINs, should be noted. LEDGINs are small molecules that inhibit lens epithelial-derived growth factor (LEDGF)/p75 cofactor for HIV-1 proviral integration [147]. LEDGINs bind dimers of HIV-1 integrase and inhibit the interaction between integrase and LEDGF/p75, which results in reduced catalytic integrase activity and relocation of residual proviral integrants out of transcription units, towards the inner nuclear component into chromatin regions increasingly associated with epigenetic marks of transcriptional silencing (e.g. H3K9me3, H3K27me3) [148, 149]. This relocation propagates a latent proviral phenotype which shows reduced activation potential by LRAs. Interestingly, LEDGINs also appear to have an inhibitory effect on late events in the HIV-1 replication cycle: Viral particles produced in the presence of the inhibitors show aberrant integrase multimerization, which leads to an impaired infection potential at several levels [148, 150]. As for dCA, future studies including trials in humans will be necessary to evaluate the potential of LEDGINs in the quest for HIV-1 remission.

Finally, observed changes in the genome-wide epigenetic profile of HIV-1-infected and bystander immune cells represent a so far unexplored but possibly interesting target for clinical approaches. This might, in particular, be the case for alterations in the cellular methylome, which have already been associated with a phenotype of ageing that might promote HIV-1 associated comorbidities [124]. A more detailed mechanistic understanding of these alterations will, however, be required, before clinical strategies, similar to the use of epi-LRAs in the ‘shock and kill’ approach, can be followed. In general, epigenetic therapies will likely play a role in future innovative approaches to HIV-1 disease, but more work will be needed to circumvent drawbacks of current epigenetic drugs, such as for example toxicity effect due to the lack of specificity [151].

## Conclusions

The cellular epigenetic machinery plays an important role in chronic infection with HIV-1. On the one hand, epigenetic mechanisms are heavily involved in regulating transcriptional silencing of the proviral-derived DNA burden. This regulation is decisive particularly since transcriptional activity of intact proviral genomes in reservoir cells results in viremic rebound with grave clinical consequences. On the other hand, infection with HIV-1 also appears to change the epigenomic landscape of infected and bystander immune cells. This signature of infection, whether directly or indirectly linked to the proviral burden, could be an important cofactor in developing HIV-1 disease-associated morbidities. Many questions remain. It will in future be necessary to broaden epigenetic studies on HIV-1 disease increasingly to primary cells and tissues of affected individuals. Improvements also need to be done on mechanistic aspects of epigenetic crosstalk, in particular in understanding how infection leads to reprogramming of the human epigenome. Furthermore, it is to date unclear whether and how epigenetic mechanisms might play a role in observed phenomena of integration site recurrence and clonal proliferation of infected cells. In view of the potential clinical importance of these phenomena, in particular for the control of reservoir size and inducibility, this aspect certainly deserves future interrogations. Despite these open questions, our current understanding of the epigenetic regulation in chronic HIV-1 infection already holds a strong indication that pharmacological agents able to interfere and modify these regulatory pathways are promising candidates in future clinical strategies for sustainable remission in HIV-1 infection.
